# A high sensitive fiber-optic strain sensor with tunable temperature sensitivity for temperature-compensation measurement

**DOI:** 10.1038/srep42430

**Published:** 2017-02-13

**Authors:** Jie Hu, Hui Huang, Min Bai, TingTing Zhan, ZhiBo Yang, Yan Yu, Bo Qu

**Affiliations:** 1 Department of Electronic Science and Technology, Dalian University of Technology, Dalian 116024, China; 2 Jordan Valley Semiconductors Ltd, Shanghai 201216, China

## Abstract

A high sensitive fiber-optic strain sensor, which consists of a cantilever, a tandem rod and a fiber collimator, was proposed. The tandem rod, which transfer the applied strain to the cantilever, was used for tuning the temperature sensitivity from −0.15 to 0.19 dB/°C via changing the length ratio of the rods. Moreover, due to the small beam divergence of the collimator, high strain sensitivity can be realized via incident-angle sensitive detection-mechanism. A strain detection-range of 1.1 × 10^3^ με (with a sensing length of 21.5 mm), a detection limit of 5.7 × 10^−3^ με, and a maximum operating frequency of 1.18 KHz were demonstrated. This sensor is promising for compensating the thermal-expansion of various target objects.

Fiber-optic strain (FOS) sensors, such as fiber Bragg grating (FBG)^1^,^2^,^3^,^4^,^5^, Fabry-Perot interferometer (FPI)^6^,^7^, Mach-Zehnder interferometer^8^, Michelson interferometer^9^, fiber mode interference^10^, fiber bending attenuation^11^, fiber Brillouin scattering^12^, fiber laser^13^, fiber long-period grating^14^, fiber loop^15^, and polarimetric^16^,^17^, etc., have attracted many research interests. The FPI sensor has demonstrated the capability of detecting sub-picostrain, which is the highest sensitivity to our knowledge, via employing Pound-Drever-Hall frequency locking technique^3^,^4^. The FBG sensor is the most widely used and commercialized FOS sensor^1^,^2^,^5^, but it has high temperature interference and can also be used for temperature sensing^5^. The commercial FBG sensor has a strain detection-range of 5 × 10^3^ με (with a sensing length of 12 mm) and a detection limit of 1.0 με^5^.

Currently, a general problem with FOS sensors is temperature interference (i.e., strain-temperature cross sensitive), and temperature sensitivity of the sensor needs to be reduced or compensated^1^,^6^,^7^,^12^,^13^,^14^,^15^,^16^. Similarly, in real strain measurement of target object (whose strain also results from both temperature variation and external force), the thermal-expansion of target object itself needs compensation, if it is necessary to obtain the strain solely induced by external force^17^. However, for different target objects (such as steel, aluminum, copper, etc), their thermal-expansion-coefficients (TECs) are different, so the FOS sensor with tunable temperature sensitivity is anticipated for compensating the TECs of different target object.

Meanwhile, the detection system of FOS sensors can be mainly categorized as wavelength- and intensity-demodulation^1^,^2^,^3^,^4^,^5^,^6^,^7^,^8^,^9^,^10^,^11^,^12^,^13^,^14^,^15^,^16^,^17^. Wavelength-demodulation technique has the unique advantage of immunity to optical power fluctuations and can be used for remote sensing^1^,^2^,^3^,^4^,^5^,^12^,^13^. While the intensity-demodulation technique features simplicity and low cost^8^,^10^,^14^,^15^, and it can be used for short distance sensing, where the transmission loss and loss variation of optical fiber is low enough. As for the optical power variation of light source, it can be compensated with an additional optical channel^7^.

In this paper, an intensity-modulation FOS sensor with tunable temperature sensitivity was proposed for the first time, which consists of an elastic cantilever, a tandem rod and a fiber collimator. The temperature sensitivity is decided by the TEC of the tandem rod, and it can be tuned in a wide range (from positive to negative) by changing the length ratio of the rods of different TECs. Moreover, due to the small beam divergence and incident-angle sensitive coupling-efficiency of the collimator, high strain sensitivity can be realized. A strain detection-range of 1.1 × 10^3^ με, a detection limit of 5.7 × 10^−3^ με, and a maximum operating frequency of 1.18 KHz were demonstrated.

## Principle

### Sensing mechanism

**Figure 1:**
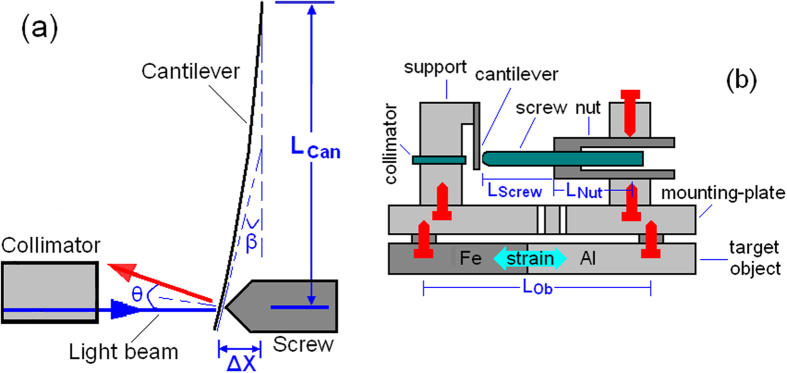
**(a)** Sensing mechanism and **(b)** Schematic structure of the FOS sensor.

Schematic structure of the FOS sensor was shown in Fig. 1. A collimator was used for both emitting and receiving light beam, which was reflected back with an incident-angle (θ) by the elastic cantilever. The strain applied on the target object can make the cantilever deflected via the tandem rod, which consists of a screw and a nut. As shown in Fig. 1(a), the incident-angle can be expressed as^18^


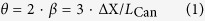


where β is the bending angle of the cantilever, L_Can_ is the free moving length (11 mm) of the cantilever, and ΔX is the strain induced cantilever-deflection.

In comparison with optical fiber, the beam divergence of fiber collimator is much smaller ( < 0.25°) 19,20. Thus, by employing fiber collimator instead of fiber, the working distance (i.e., the distance between the collimator and cantilever) can be increased from micrometer to centimeter level, which increases the cantilever deflection-range (i.e., strain detection-range) and also decreases the manufacturing difficulty of the sensor.

Moreover, owning to the small beam divergence, coupling efficiency of the collimator is very sensitive to the incident-angle θ rather than the axial- or lateral-offset^19^,^20^. The θ induced coupling loss can be expressed as^20^


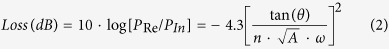


where P_In_ and P_Re_ is the incident and received optical power of the collimator, n is the refractive index (1.59) of the collimator, A^1/2^ is the gradient constant (0.53 mm^−1^) of the collimator, ω is the mode-field radius (5.2 μm) of the single-mode fiber.

### Temperature compensation via tuning the TEC of the tandem rod

As shown in Fig. 1, strain of the target object, which results from both thermal-expansion and external-force, can induce cantilever-deflection via the tandem rod. Thus, the deflection (ΔX) can be expressed as


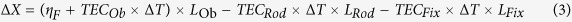


Where η_F_ is external-force induced strain on the target object, TEC_Ob_ and L_Ob_ (21.5 mm) is the TEC and sensing length of the target object, TEC_Rod_ and L_Rod_ (16.5 mm) is the TEC and length of the tandem rod, TEC_Fix_ and L_Fix_ (5.5 mm) is the TEC and length of the fixing supports (used for fixing the cantilever and the tandem rod), respectively, and ΔT is the temperature variation.

For different target objects, if their TEC_Ob_ is compensated by adjusting the TEC_Rod_ (i.e., TEC_Rod_ × L_Rod_ = TEC_Ob_ × L_Ob_ − TEC_Fix_ × L_Fix_), the cantilever deflection can be solely decided by the external force without temperature interference (i.e., ΔX = η_F_ × L_Ob_).

As shown in Fig. 1, the tandem rod is constructed by an adjustable-screw and a sleeve-nut of different TECs (i.e., TEC_Screw_ and TEC_Nut_), so the TEC_Rod_ can be tuned between TEC_Screw_ and TEC_Nut_ by changing the length ratio of the screw and the nut. The TEC_Rod_ can be expressed as


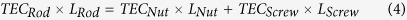


Where L_Rod_ = L_Nut_ + L_Screw_, and L_Screw_ and L_Nut_ is the length of the screw and the nut, respectively.

### Device Fabrication and Measurement

**Figure 2:**
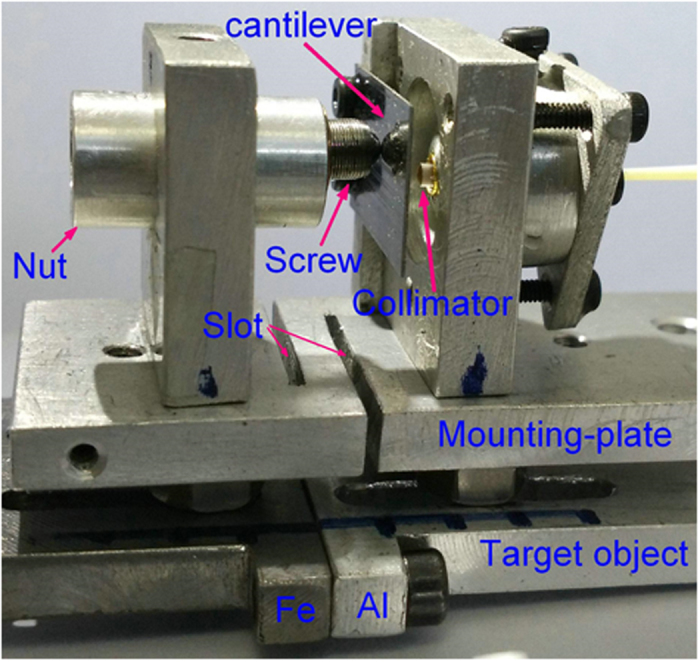
The optical photograph of the fabricated sensor.

**Figure 3:**
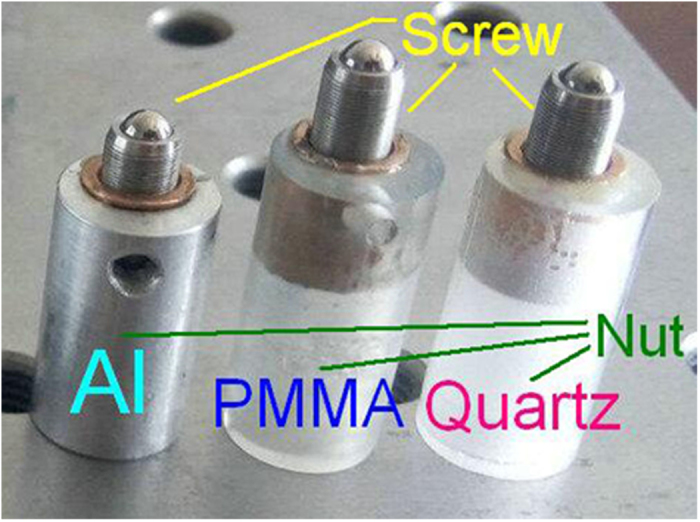
The optical photograph of the tandem rod.

As shown in Fig. 1 and Fig. 2, The FOS sensor consists of an elastic cantilever (a 15 mm long and 0.4 mm thick spring-steel slice with one polished surface for reflecting light beam), a tandem rod and an optical fiber collimator (with a diameter of 3 mm and a working distance of 8 mm). As shown in Fig. 3, the tandem rod is constructed by a stainless-steel adjuster-screw and a sleeve-nut (made from Al, PMMA, or quartz). The screw is fixed inside the nut, and its length outside the nut can be precisely tuned. Thus, TEC_Rod_ can be tuned between the TECs of screw (stainless-steel) and nut (such as Al, PMMA or quartz).

Fabrication of the FOS sensor was carried out in the following steps (Fig. 1(b) and Fig. 2): (1) the cantilever was fixed on one side of an Al-alloy-support (AAS), and the collimator was fixed on the other side of the AAS after it was precisely adjusted for vertical alignment to the cantilever; (2) the tandem rod was fixed on another AAS, (3) then these two AAS were fixed on an Al mounting-plate, which can be stretched/compressed easily due to its two open slots (Fig. 2). For improving the sensor stability, each part of the FOS sensor was fixed without using glue. Optical image of the fabricated sensor was shown in Fig. 2.

The detection system consists of a 1550 nm laser (Opeak Corp., DFB-LSM-1550) and a photodetector (Thorlabs PDB450C), which were connected with the fiber collimator. The output optical power of the laser was kept constant at 2.0 mW.

**Figure 4:**
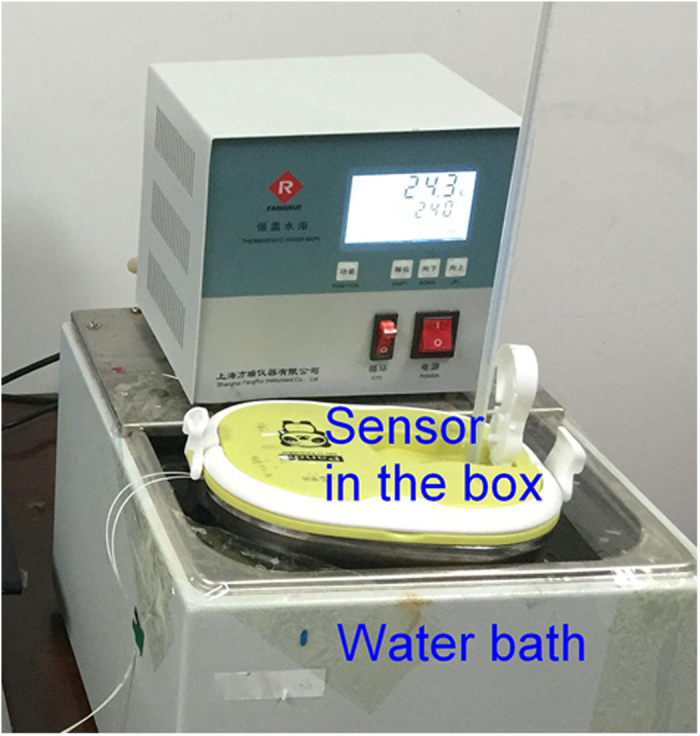
Experiment setup for temperature sensitivity measurement.

For temperature sensitivity measurement, the target object is a joined Al-Fe plate with a sensing length (L_Ob_) of 21.5 mm, which is a 10 mm long Fe plate (TEC of 12.0 × 10^−6^/K) joined with a 11.5 mm long Al plate (TEC of 22.2 × 10^−6^/K) (Fig. 2). So the average TEC of the target object is 17.5 × 10^−6^/K. As shown in Fig. 4, the target object fixed with the sensor was sealed in a stainless-steel box. Then the box was put into a water-bath for tuning the temperature. The detection system was kept at a constant temperature of 26 °C.

**Figure 5:**
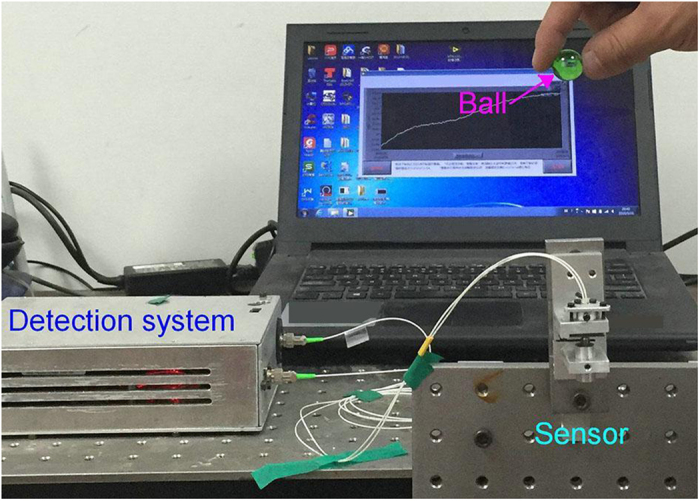
Experiment setup for response speed measurement.

**Figure 6:**
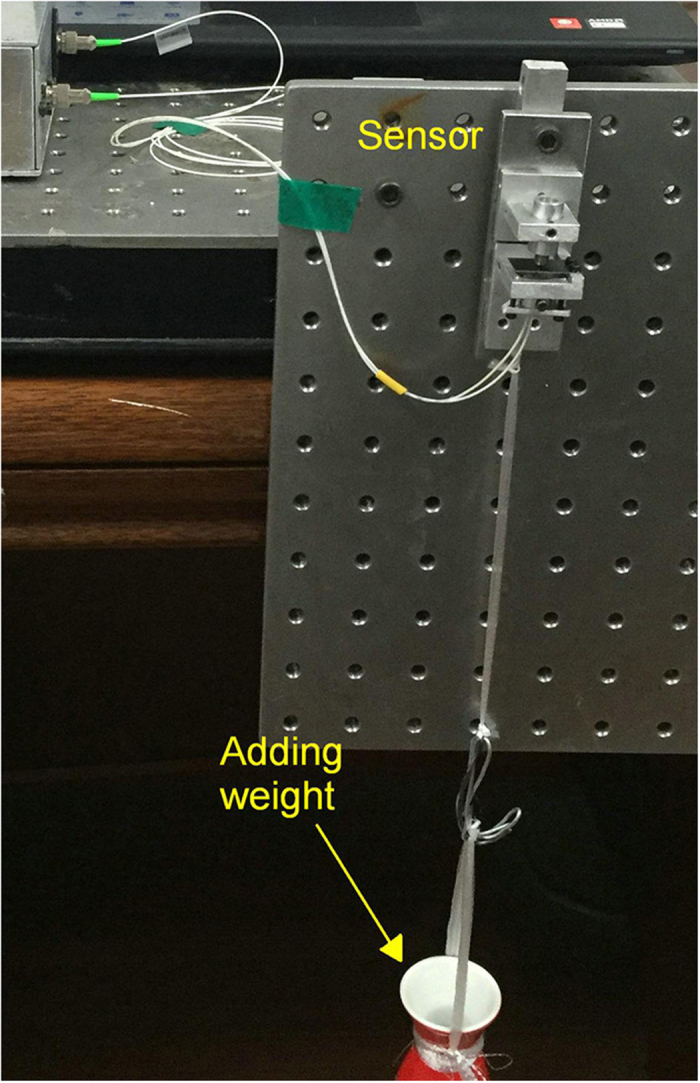
Experiment setup for the measurement of external-force induced strain.

For strain measurement, the target object, which is a single Fe plate with a cross section of 2.5 mm × 20 mm and a sensing length (L_Ob_) of 36 mm, was fixed vertically with one-end anchored and another-end free. For response speed measurement (Fig. 5), the free top-end of the target object was hit by a falling glass ball, and the data was collected with a sampling rate of 46 KHz. As for the measurement of external-force induced strain (η_F_), the free bottom-end of the target object was hanged with a bottle (Fig. 6). Weight of the bottle was increased successively by added a fixed volume of water. If the adding weight is larger than 250 g, the bottle was replaced with Fe blocks. The strain (η_F_) can be expressed as^18^


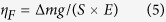


where S (50 mm^2^) is the cross-section area of the Fe plate, E is the Fe Young modulus of 2.1 × 10^5^ N/mm^2^, and Δmg is the adding weight.

## Results and Discussion

**Figure 7:**
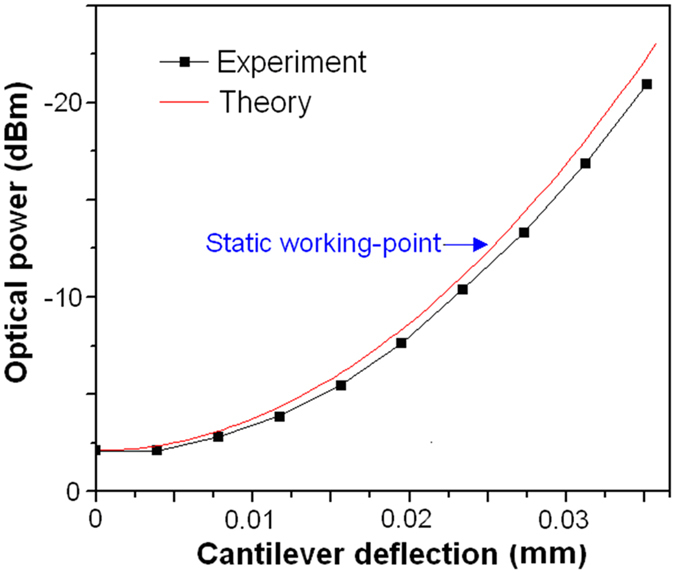
Relationship between the received optical power and the cantilever deflection.

As shown in Fig. 7, relationship between the received optical power (P_Re_) of the collimator and the cantilever deflection (ΔX) was obtained by using Eqs 1 and 2, and it was measured by precise tuning the adjuster-screw of the tandem rod. It can be seen that a deflection range between 0.01 mm and 0.035 mm can be obtained, which corresponds to a strain detection-range of 1.1 × 10^3^ με with a L_Ob_ of 21.5 mm. For symmetric measurement of both tensile and compressive strain, a static working-point with an off-set deflection of 0.025 mm and a corresponding off-set optical power of −12.3 dBm was chose (Fig. 7).

**Figure 8:**
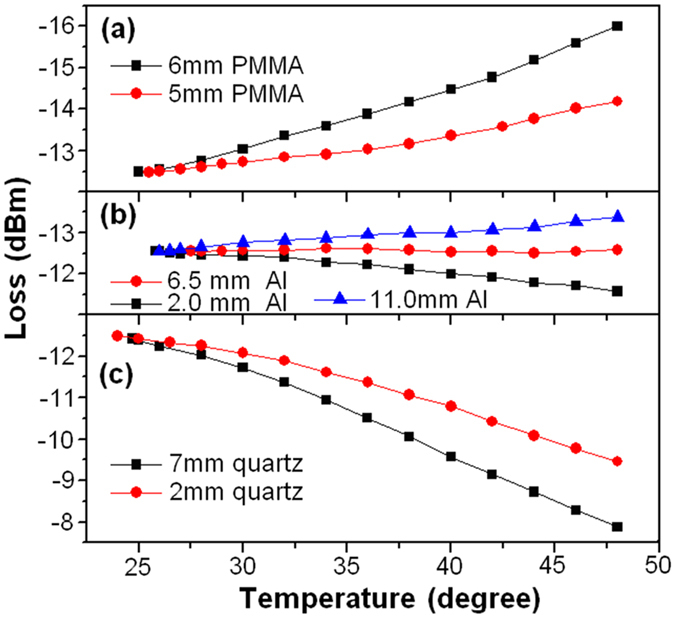
Measured temperature sensitivity of the FOS sensor with (**a**) PMMA nut, (**b**) Al nut, and (**c**) quartz nut.

Figure 8 shows the measured temperature sensitivity of the FOS sensor. According to Eqs 3 and 4, the temperature sensitivity can be tuned by changing the length ratio of the sleeve-nut and the adjustable-screw. With a fixed length of the tandem rod (L_Rod_ = L_Nut_ + L_Screw_ = 13.6 mm), the temperature sensitivity is −0.15 and −0.07 dB/°C for 6 mm and 5 mm long PMMA nut (Fig. 8(a)), is −0.03, 0.0008 and 0.04 dB/°C for 11.0 mm, 6.5 mm and 2.0 mm long Al nut (Fig. 8(b)), and is 0.12 and 0.19 dB/°C for 2 mm and 7 mm long quartz nut (Fig. 8(c)), respectively.

It can be seen that the temperature sensitivity can be tuned in a wide range from positive to negative by increasing the TEC_Nut_ (TEC_PMMA_ > TEC_Al_ > TEC_quartz_), because larger TEC_Nut_ would increase the cantilever deflection (ΔX) and then result in larger coupling loss of the collimator (Fig. 1). In this case, for the target object made from joined Fe-Al plate, temperature insensitivity can be obtained with a 6.5 mm long Al nut.

**Figure 9:**
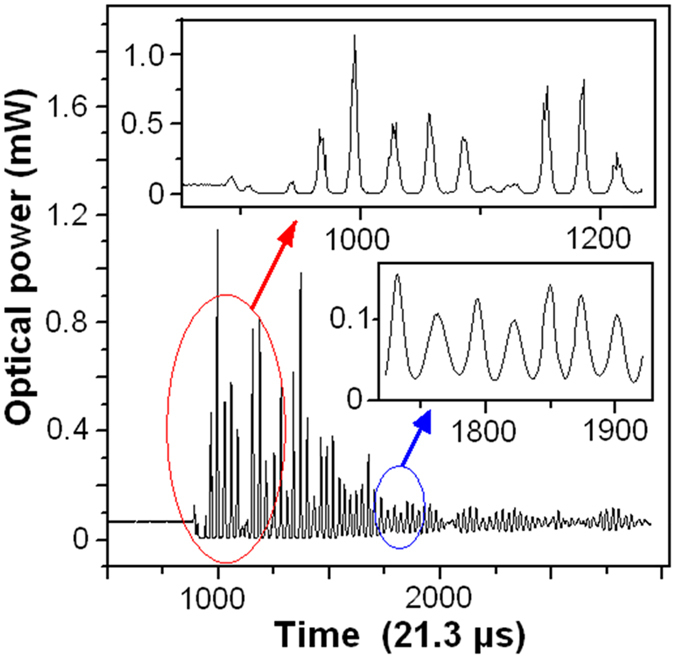
Measurement results of dynamic response of the FOS sensor.

Figure 9 shows the dynamic response of the FOS sensor hit by the glass ball. It can be seen that the sensor vibrates at a resonant frequency of 1.67 KHz. Thus, the maximum operating frequency of the sensor is 1.18 KHz by multiplying the resonant frequency with a factor of 0.707.

**Figure 10:**
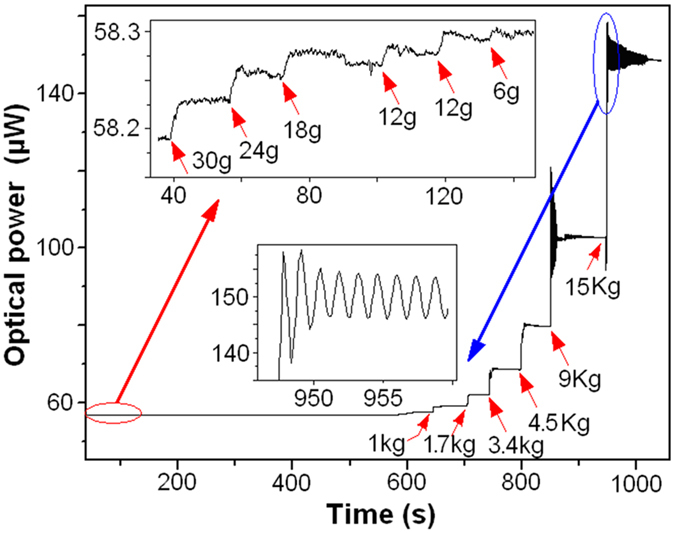
Measurement results of the strain induced by adding weight.

**Figure 11:**
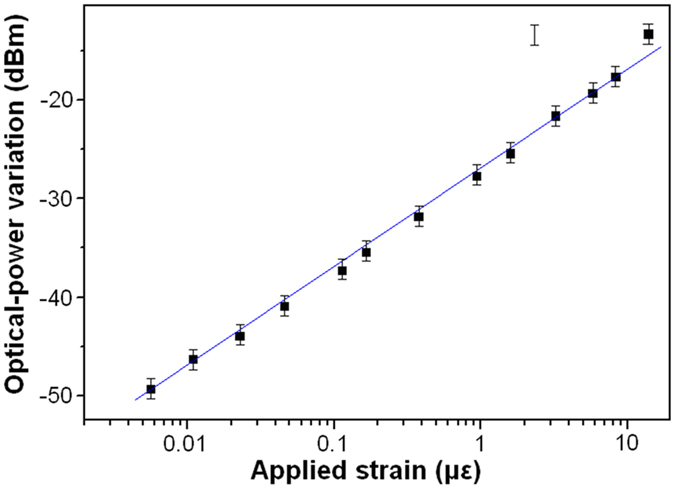
The relationship between the measured optical-power variation and applied strain.

Figure 10 shows the measurement results of external-force induced strain, which was generated by adding weights. As shown by the inset indicated by red arrow, an adding weight as low as 6.0 g is detectable, which corresponds to a detection limit of 5.7 × 10^−3^ με (Eq. 5). The detection limit can be further improved by employing lower noise laser and photodetector (the noise is about 7 nW in this case). As shown by the inset indicated by blue arrow, when the adding weight is larger than 9 kg, signal oscillation resulted from the pendulum of the Fe blocks was observed. Figure 11 plots the relationship between the measured optical-power variation and applied strain.

For the FOS sensor, the detection-range (1.1 × 10^3^ με) is smaller than that of commercial FBG sensor (5 × 10^3^ με)^5^, and the detection limit (5.7 × 10^−3^ με) is much better than that of commercial FBG sensor (1.0 με)^5^. In the future, the minimization of the sensor will be carried out by using MEMS technology, and the laser can be replaced by LED for low cost detection-system. If the ball lensed fiber, which has a working distance as long as 3 mm (BL-5, WT&T Inc., www.wttechnology.com), is employed as micro-collimator, high temperature, compact and light weight sensor can be realized.

## Conclusion

A high sensitive FOS sensor with tunable temperature sensitivity was proposed for the first time. The sensor simply consists of an elastic cantilever, a tandem rod and a fiber collimator. The tandem rod, which transfer the strain to the cantilever, was used to tune the temperature sensitivity in a wide range (from −0.15 to 0.19 dB/°C) by changing the TEC and length ratio of the screw/nut. Moreover, due to the small beam divergence of collimator, high strain sensitivity can be realized via incident-angle sensitive detection-mechanism. A strain detection-range of 1.1 × 10^3^ με (with a sensing length of 21.5 mm), a detection limit of 5.7 × 10^−3^ με, and a maximum operating frequency of 1.18 KHz were demonstrated. This sensor is promising for compensating the thermal-expansion of target objects made from various materials.

## Additional Information

**How to cite this article**: Hu, J. *et al*. A high sensitive fiber-optic strain sensor with tunable temperature sensitivity for temperature-compensation measurement. *Sci. Rep.* **7**, 42430; doi: 10.1038/srep42430 (2017).

**Publisher's note:** Springer Nature remains neutral with regard to jurisdictional claims in published maps and institutional affiliations.
